# Transforming Growth Factor: β Signaling Is Essential for Limb Regeneration in Axolotls

**DOI:** 10.1371/journal.pone.0001227

**Published:** 2007-11-28

**Authors:** Mathieu Lévesque, Samuel Gatien, Kenneth Finnson, Sophie Desmeules, Éric Villiard, Mireille Pilote, Anie Philip, Stéphane Roy

**Affiliations:** 1 Department of Biochemistry, Université de Montréal, Montréal, Québec, Canada; 2 Faculty of Dentistry, Université de Montréal, Montréal, Québec, Canada; 3 Department of Surgery, Faculty of Medicine, McGill University, Montréal, Québec, Canada; Ordway Research Institute, United States of America

## Abstract

Axolotls (urodele amphibians) have the unique ability, among vertebrates, to perfectly regenerate many parts of their body including limbs, tail, jaw and spinal cord following injury or amputation. The axolotl limb is the most widely used structure as an experimental model to study tissue regeneration. The process is well characterized, requiring multiple cellular and molecular mechanisms. The preparation phase represents the first part of the regeneration process which includes wound healing, cellular migration, dedifferentiation and proliferation. The redevelopment phase represents the second part when dedifferentiated cells stop proliferating and redifferentiate to give rise to all missing structures. In the axolotl, when a limb is amputated, the missing or wounded part is regenerated perfectly without scar formation between the stump and the regenerated structure. Multiple authors have recently highlighted the similarities between the early phases of mammalian wound healing and urodele limb regeneration. In mammals, one very important family of growth factors implicated in the control of almost all aspects of wound healing is the transforming growth factor-beta family (TGF-β). In the present study, the full length sequence of the axolotl TGF-β1 cDNA was isolated. The spatio-temporal expression pattern of TGF-β1 in regenerating limbs shows that this gene is up-regulated during the preparation phase of regeneration. Our results also demonstrate the presence of multiple components of the TGF-β signaling machinery in axolotl cells. By using a specific pharmacological inhibitor of TGF-β type I receptor, SB-431542, we show that TGF-β signaling is required for axolotl limb regeneration. Treatment of regenerating limbs with SB-431542 reveals that cellular proliferation during limb regeneration as well as the expression of genes directly dependent on TGF-β signaling are down-regulated. These data directly implicate TGF-β signaling in the initiation and control of the regeneration process in axolotls.

## Introduction

Urodele amphibians, such as the axolotl (*Ambystoma mexicanum*), have the unique ability, among vertebrates, to perfectly regenerate many parts of their body throughout their life. Among the complex structures that can be regenerated in the axolotl, the limb is the most widely studied [1]–[3]. Limb regeneration represents an elaborate process in which wound healing, cellular dedifferentiation, tissue remodeling and patterning occur to replace the amputated appendage. Understanding urodele limb regeneration could be helpful in the design and development of novel therapies in regenerative medicine. It is therefore interesting to highlight the fact that following limb amputation, the axolotl will regenerate its limb without any residual scar between the stump and the regenerated structure [1], [2]. The vast majority of adult vertebrates including mammals, birds, fishes, anuran amphibians and reptiles are incapable of regenerating complex structures such as limbs or tail. In those species, wound healing results in scarring. Thus, scarring, or fibrosis, seems to be a universal response to wounding that spans the 3 layers (epidermis, dermis and hypodermis) of the skin in vertebrates. The similarities between regeneration and wound healing have been discussed by many authors [2], [4]–[6] and it has become apparent that a proper understanding of both similarities and differences is essential to help us better comprehend how regeneration is achieved in urodeles but not in mammals.

Axolotl limb regeneration is considered by many to be divided in two main phases [2], [7], [8]. The first phase is referred to as the preparation phase and begins immediately following amputation with the formation of a wound epithelium (WE) over the amputation plane. Cellular dedifferentiation and migration, which will eventually lead to the formation of a regeneration blastema, also take place in this phase. In the second phase of limb regeneration, referred to as the redevelopment phase, blastema cells stop proliferating and start to redifferentiate to regenerate the lost part [1], [8].

The preparation phase of regeneration in urodeles shares many similarities with wound healing in mammals [2], [9], [10]. Both regeneration and wound healing are triggered by a trauma (amputation or wounding) which is followed by the up-regulation of many stress signals, inflammation and the formation of a blood clot [5], [9]. These events all occur within minutes after the trauma and are followed shortly by the formation of a wound epithelium [1], [11]–[13]. The WE is usually formed within 2–6 hours post-amputation in urodeles and between 12–48 hours post-wounding in mammals [5], [10], [14]. If formation of the WE is prevented, regeneration does not proceed and wound healing is retarded significantly [1], [10], [11], [15], [16]. During the preparation phase, following WE formation, there is extensive remodelling of the extracellular matrix (ECM) through the action of matrix metalloproteinases (MMP) and tissue inhibitors of metalloproteinases as for wound healing in mammals [17]–[21]. Inhibitors of MMPs, such as GM6001, have been shown to inhibit limb regeneration and to cause the appearance of a scar-like layer of skin with collagen deposits on the stump of amputated limbs [17].

As stated above, many cellular and physiological processes are common to both axolotl limb regeneration and mammalian wound healing. An important regulator of many of the aforementioned events during wound healing is the transforming growth factor-beta (TGF-β) signaling pathway. The TGF-β super-family contains more than 30 structurally related growth and differentiation factors including TGF-βs, BMPs, activins, inhibins, and GDFs [22], [23]. TGF-β1 is the most studied member of this family for its roles in wound healing processes and immune response in mammals [24]–[28]. TGF-β1 signals through two transmembrane serine/threonine kinase receptors [TGF-β type I receptor (also called ALK 5) and TGF-β type II receptor] that phosphorylate the SMADs (SMAD 2 and 3) which activate target genes in the nucleus. TGF-β1 has been shown to induce proliferation of skin fibroblasts and to promote the migration of fibroblasts and keratinocytes during wound healing [29]–[33]. The participation of dermal fibroblasts in salamander limb regeneration was first reported in 1954 and later confirmed by other groups [1], [34]–[36]. TGF-β1 also activates many target genes implicated in wound healing and ECM production including connective tissue growth factor (CTGF) and fibronectin, which were both shown to be expressed in salamander regenerating tissues [15], [28], [37].

By combining PCR and cDNA library screening techniques, we isolated the full length cDNA sequence of the axolotl TGF-β1. We demonstrate that the TGF-β1 mRNA is up-regulated during the preparation phase of limb regeneration and then down-regulated during the redevelopment phase. These data are suggestive of a role for TGF-β1 in the initiation and control of the regeneration process. To address the requirement of TGF-β during regeneration, a specific inhibitor of TGF-β signaling, SB-431542, was used [38]. The ease of administration of small pharmacological inhibitors makes them powerful tools to address the role of specific signaling/molecular pathways, such as the TGF-β pathway, in complex *in vivo* physiological processes such as limb regeneration. Recent publications on tissue regeneration present interesting results using such inhibitors [17], [39]. Many studies have used SB-431542 to specifically block TGF-β signaling and TGF-β1 mediated effects [38], [40]–[44]. Our results show that SB-431542 blocks limb regeneration in axolotls thus suggesting that TGF-β signaling is essential for limb regeneration.

## Results

### Identification of axolotl TGF-β1

**Figure 1:**
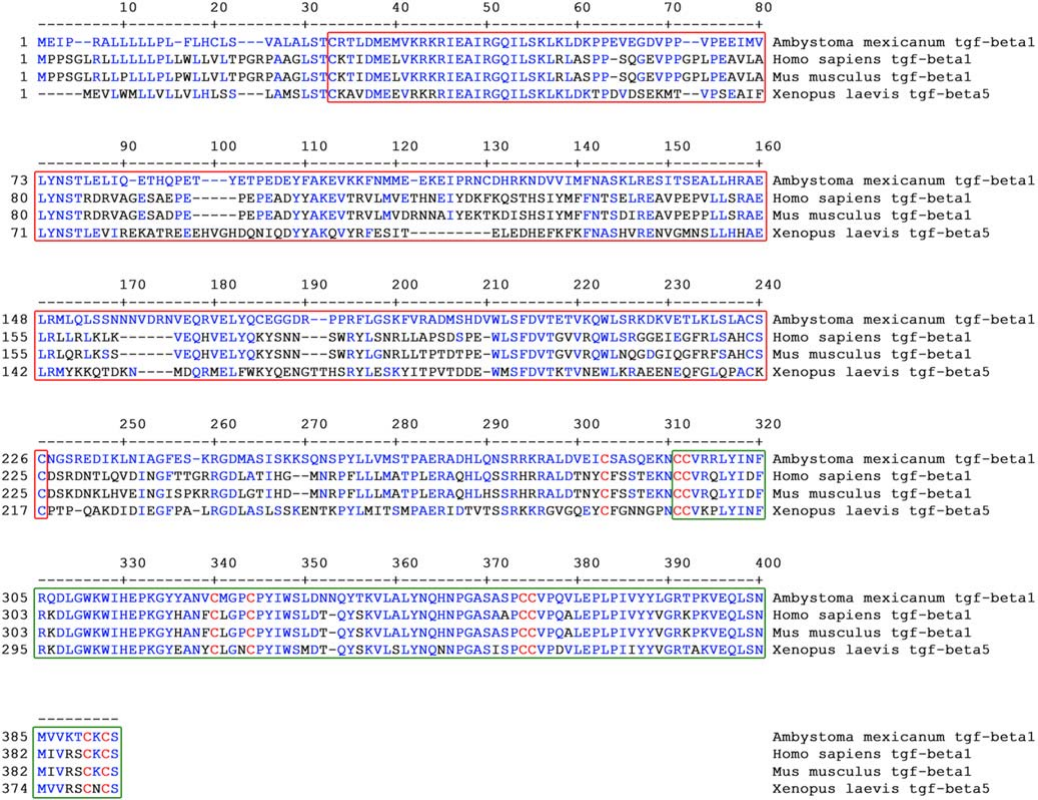
Axolotl TGF-β1 protein sequence and domains. Alignment of axolotl (*Ambystoma mexicanum*) TGF-β1 amino acid sequence with TGF-β1 sequences of human (*Homo sapiens*), mouse (*Mus musculus*) and TGF-β5 of Xenopus (*Xenopus laevis*). In blue are residues of the axolotl sequence that are conserved in the other three sequences. In red are the 9 conserved cysteine residues found in every TGF-β1 sequence. The red box identifies the pro-domain of the protein. The green box identifies the mature TGF-β domain of the protein.

Of the three mammalian TGF-β isoforms (i.e. TGF-β1, TGF-β2 and TGF-β3), TGF-β1 is recognized as the most important in events related to wound healing processes and scar formation [27], [45], [46]. By using a combination of cDNA library screening, RT-PCR and RACE-PCR, a 1179 nucleotide cDNA sequence corresponding to the axolotl TGF-β1 was isolated (GenBank accession number EU147783). This fragment encodes a protein of 393 amino acids. As observed in other species, the sequence contains a pro-domain and a TGF-β domain (Fig. 1). The pro-domain of the axolotl protein aligns with the pro-domains of the human and mouse proteins as 43% of the amino acid residues are identical between the three sequences. The pro-domain, also called the LAP (latency associated peptide, which is cleaved but remains associated with the TGF-β domain until activation [47]), is identified by a red box (Fig. 1). The mature TGF-β domain of the axolotl protein (identified by a green box, Fig. 1) is well conserved with 85% identity in amino acids compared to the human and mouse domains. The axolotl TGF-β1 sequence was also compared with *Xenopus laevis* TGF-β5. The axolotl TGF-β1 protein has 44% residue identity in the pro-domain and 80% identity within the TGF-β domain of *Xenopus laevis* TGF-β5, which is comparable with that of the human and mouse TGF-β1. In addition, nine conserved cysteines which are considered essential for TGF-β dimerization and transport were identified in the TGF-β domain of the axolotl sequence (cysteines are identified in red, Fig. 1) [48].

### Expression of TGF-β1 mRNA during axolotl limb regeneration

**Figure 2:**
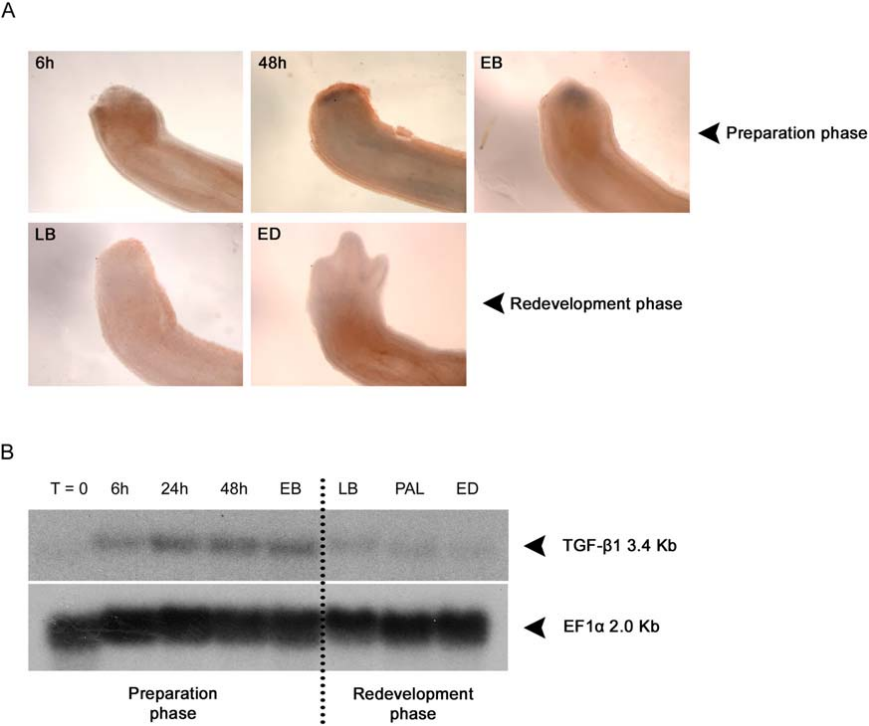
Expression of TGF-β1 during axolotl limb regeneration. A) Expression of TGF-β1 determined by whole-mount *in situ* hybridization in axolotl forelimbs. Limbs were amputated distally through radius/ulna. Samples were fixed at various times after amputation: 6 hours (6 h), 48 hours (48 h), early bud (EB), late bud (LB) and early differentiation (ED) stage. TGF-β1 expression is found as a dark purple precipitate. The 3 stages on the upper row are part of the preparation phase of limb regeneration. The 2 stages on the lower row are part of the redevelopment phase of limb regeneration. B) Northern blot showing expression of TGF-β1 at various stages of forelimb regeneration. TGF-β1 was detected as a 3.4 Kb transcript. RNA was extracted from regenerating blastemas at various times after amputation: 6 hours (6 h), 24 hours (24 h), 48 hours (48 h), early bud (EB), late bud (LB), palette (PAL) and early differentiation (ED) stage. T = 0: RNA was extracted from an unamputated mature limb. The dotted line marks a distinction between regeneration stages included in the preparation phase and those included in the redevelopment phase of regeneration.

Whole-mount *in situ* hybridization was performed to verify if TGF-β1 was expressed during the regeneration process. Figure 2 presents the spatio-temporal expression pattern of the TGF-β1 mRNA during limb regeneration. Expression of TGF-β1 was detected as early as 6 hours post-amputation. Although the expression at 6 hours post-amputation is hardly visible in whole-mount samples (Fig. 2 A), it was detectable by Northern blot analysis (Fig. 2 B). At 48 hours post-amputation, a strong up-regulation of TGF-β1 mRNA was detected in whole-mounts (Fig. 2 A). A few days later, at the EB stage, TGF-β1 was still strongly expressed and covered most of the blastema. The first 3 time points of Figure 2 A are part of the preparation phase of regeneration. During the redevelopment phase of limb regeneration, expression of TGF-β1 mRNA was very diffuse and barely visible in the blastema from late bud to early differentiation stage, in whole-mount samples (Fig. 2 A). Very week bands could be detected on the Northern blots at these stages (Fig. 2 B). Each lane on the Northern blot represents a pool of mRNA collected from about 30 regenerating blastemas per stage, thus making this technique more sensitive than whole-mount *in situ* hybridizations which are individual blastema. Together, these results show that TGF-β1 mRNA is expressed and regulated during limb regeneration in the axolotl.

### Presence of TGF-β receptors in axolotl cells

**Figure 3:**
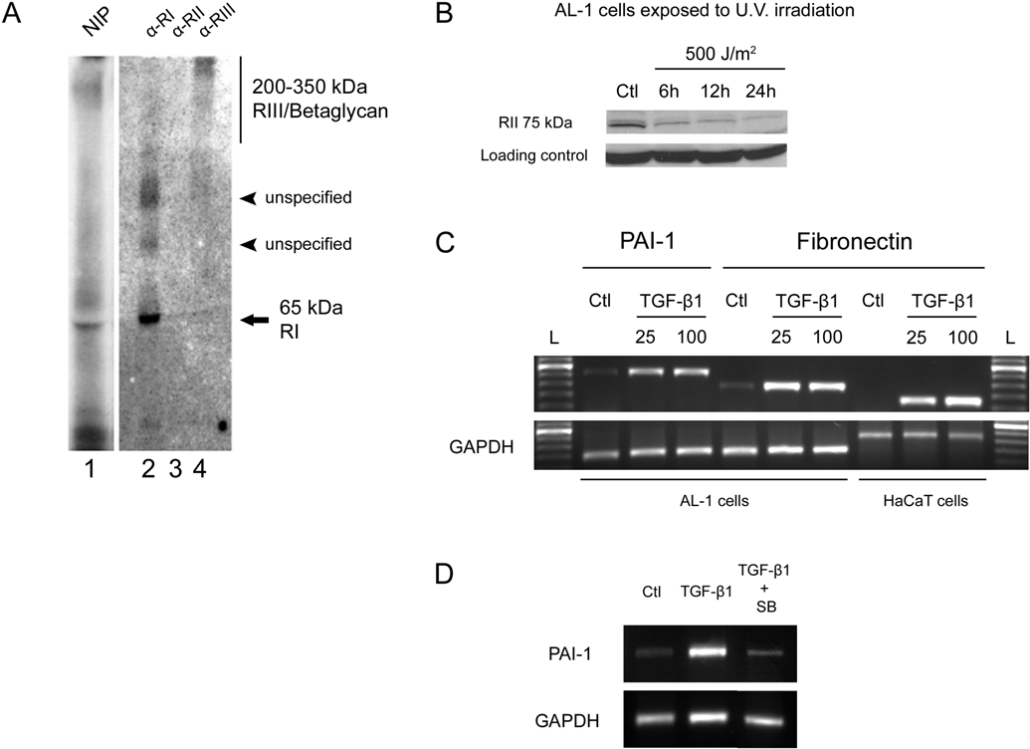
Detection of TGF-β receptors and target genes in axolotl cells. A) Presence of TGF-β receptors after affinity labeling of axolotl AL-1 cell line with [^125^I]-TGF-β1. Lane 1: (NIP): Cell lysates were not immuno-precipitated. A band corresponding to TGF-β RI around 65 kDa can be observed. Lane 2: Proteins were immuno-precipitated with anti-TGF-β RI antibody. A band corresponding to TGF-β RI at 65 kDa is also observed in this lane. Two other bands were observed in this lane. These bands correspond to unspecified proteins that co-immuno-precipitated with TGF-β RI. Lane 3: Proteins were immuno-precipitated with anti-TGF-β RII antibody. No band was detected. Lane 4: Proteins were immuno-precipitated with anti-TGF-β RIII/betaglycan antibody. A diffuse band of high molecular weight (200–350 kDa) corresponding to TGF-β RIII/betaglycan was detected. B) Western blot experiment showing the presence of TGF-β RII in axolotl AL-1 cells. Ctl lane represents TGF-β RII protein expression in control axolotl cells not exposed to U.V. light. Other lanes present diminished expression of TGF-β RII in axolotl cells exposed to 500 J/m^2^ U.V. and collected after 6 h, 12 h and 24 h. Expression of TGF-β RII in control cells at 6 h, 12 h and 24 h was stable (data not shown). Loading control (tubulin) confirms equal loading of protein samples. C) RT-PCR results of PAI-1 and fibronectin expression in AL-1 cells after stimulation with human recombinant TGF-β1. Strong up-regulation of both genes was observed after stimulating cells with 25 or 100 pM TGF-β1. Up-regulation was detected 3 h and 72 h after stimulation for PAI-1 and fibronectin respectively. The human keratinocyte cell line (HaCaT) was used as a positive control for fibronectin induction at 72 h. Ctl: control cells treated only with 4 mM HCl, 0,1% bovine serum albumin buffer (TGF-β1 carrier solution) . L: 100 base-pair DNA ladder with the most intense band at 600 bp. GAPDH was used as a control gene. D) RT-PCR results showing PAI-1 expression in AL-1 cells after stimulation with DMSO, human recombinant TGF-β1 and human recombinant TGF-β1 in the presence of SB-431542. The TGF-β1 stimulated expression of PAI-1 was significantly inhibited by SB-431542.

The presence of the TGF-β signaling machinery such as the type I, II and III receptors were determined in our model using standard biochemical approaches. First, affinity labeling using [^125^I]-labeled human TGF-β1 was performed on the AL-1 cell line, a fibroblast cell line derived from the dermis of an axolotl limb [49]. This technique involves covalent cross-linking of radioactively labeled TGF-β1 to the receptors at the surface of the cell membrane [50]. After cell lysis, the three main receptors for TGF-β signaling: TGF-β type I receptor, TGF-β type II receptor and TGF-β type III receptor, also called betaglycan [51], were immuno-precipitated. Figure 3 A presents the results of the immuno-precipitations. In lane 2, a band of 65 kDa corresponding to TGF-β type I receptor can be observed. Subtracting the molecular weight of the cross-linked monomeric TGF-β1 (12,5 kDa) results in the expected size for TGF-β type I receptor, 53 kDa [52]. Two other bands corresponding to unspecified proteins can also be seen in lane 1. It was not possible to immuno-precipitate any labeled proteins with the anti-TGF-β type II receptor antibody used (Fig. 3 A, lane 3). This could simply be due to non-immuno-reactivity of the antibody with the axolotl receptor under these conditions. The band in lane 4 corresponds to betaglycan, its high molecular weight (200–350 kDa) and diffuse migration pattern are identical to that of betaglycan previously shown in mammalian cell types [53], [54]. Having not succeeded in identifying TGF-β type II receptor by immuno-precipitation, an alternative approach was used to determine whether or not it was present. Western blot analysis was performed on AL-1 cell protein extracts and a band of the expected size (75 kDa) was observed (Fig. 3 B). In order to confirm that this band was the type II receptor, the ability of UV irradiation to reduce the expression of the specific band detected on the Western blot was tested. A previous study had reported that UV irradiation of human skin fibroblasts and mink lung epithelial cells caused a decrease in TGF-β type II receptor protein expression [55], [56]. When axolotls cells were irradiated with high levels of UVs (500 J/m^2^), the expression of the band corresponding to the TGF-β type II receptor protein was down-regulated, as previously described [55], [56] (Fig. 3 B). These results demonstrate that the two main signaling TGF-β receptors (type I and II) and one important accessory receptor (betaglycan) are present in limb derived axolotl cells. Together with the identification of cDNAs encoding SMAD proteins in the axolotl genome by the group of Voss [57], our data suggest this species possesses the whole TGF-β signal transduction machinery.

### Activation of TGF-β1 target genes in axolotl cells

To verify that the TGF-β signaling cascade is functional in axolotl cells, AL-1 cells were treated with human recombinant TGF-β1 protein and checked for target gene activation. The expression of two TGF-β1 target genes was examined: plasminogen activator inhibitor-1 (PAI-1), an immediate-early gene, and fibronectin, a late response gene. Treatment of axolotl cells with 25 or 100 pM TGF-β1 resulted in the up-regulation of PAI-1 expression 3 hours after stimulation (Fig. 3 C). This correlates with the early expression observed in other fibroblast cell lines [58]. The stimulation of axolotl cells with TGF-β1 also resulted in a strong up-regulation of fibronectin expression after 72 hours of treatment (Fig. 3 C), as shown in a previous study in human dermal fibroblasts [59]. To confirm our results, human keratinocytes HaCaT cells were used as a positive control and checked for fibronectin induction after 72 hours of treatment with TGF-β1. As previously published, HaCaT cells responded to TGF-β1 by an up-regulation of fibronectin expression (Fig. 3 C) [60]. These results, combined with the presence of the main TGF-β receptors in axolotl cells, confirm that the TGF-β signaling machinery efficiently transduces signal to the nucleus in axolotl cells. The ability of SB-431542 to block TGF-β1 target gene activation was tested in AL-1 cells. At a final concentration of 25 µM, SB-431542 efficiently blocked the TGF-β1-mediated up-regulation of PAI-1 (Fig. 3 D).

### Inhibition of axolotl limb regeneration by SB-431542

**Figure 4:**
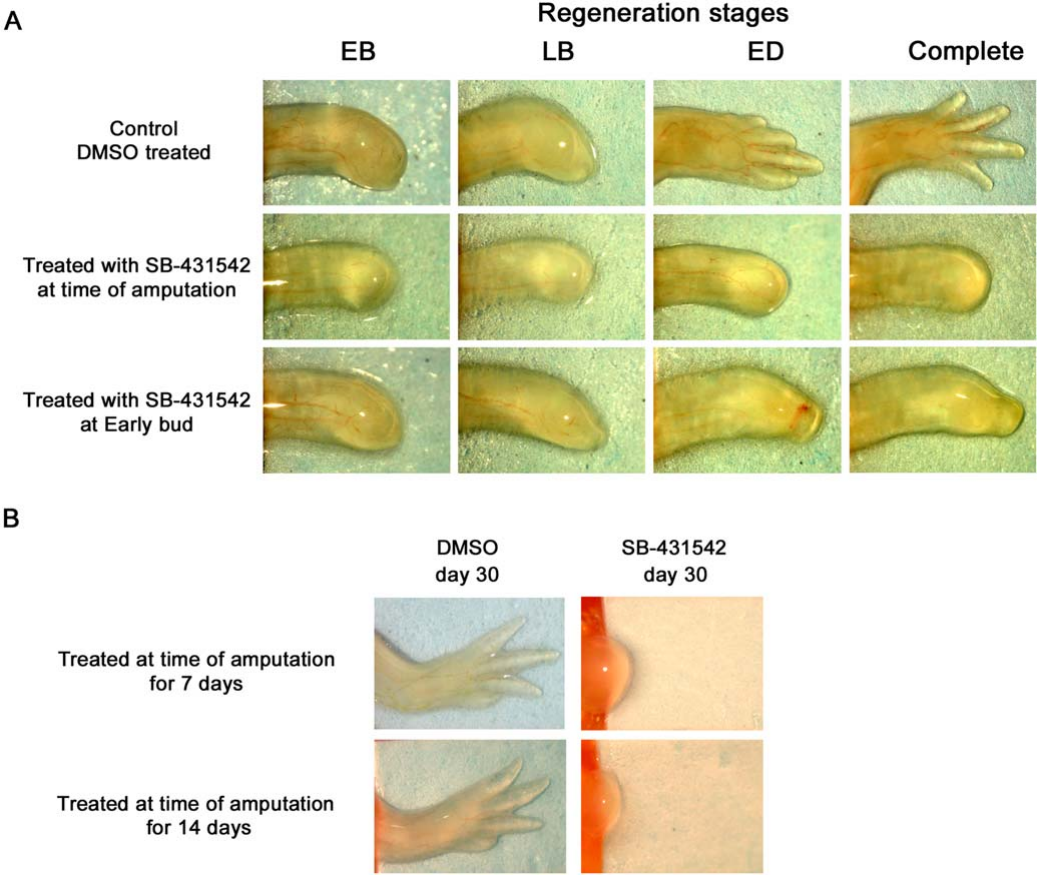
Inhibition of limb regeneration by SB-431542. A) Morphology of axolotl regenerating forelimbs treated with SB-431542. Top row: morphology of a regenerating control limb treated with DMSO. All limbs were distally amputated on the same day. Limbs were photographed when control limbs reached each of the stages indicated on top: early bud (EB), late bud (LB), early differentiation (ED). Complete: regeneration of control limb ended after 30 days. Middle row: morphology of an axolotl limb treated with 25 µM SB-431542 from the time of amputation until the control had regenerated (30 days). Complete inhibition of regeneration and absence of blastema formation were observed in these limbs. Bottom row: morphology of an axolotl limb treated with 25 µM SB-431542 from early bud stage until the control had regenerated. Growth of the blastema was observed in these limbs until it resembled a late bud blastema. B) Inhibition of regeneration with SB-431542 cannot be rescued after 7 or 14 days of treatment. In this panel, limbs were treated with DMSO or SB-431542 from the moment of amputation for the first 7 or 14 days only. Results show that limbs treated with SB-431542 for the first 7 or 14 days after amputation do not regenerate even if treatment was stopped. Control limbs treated for 7 and 14 days with DMSO regenerated normally.

Many studies have shown that SB-431542 can inhibit TGF-β signaling by binding to TGF-β type I receptor thus preventing SMAD phosphorylation [38], [40]–[43]. SB-431542 is highly specific as it blocks TGF-β signaling, but not BMP or SMAD-independent TGF-β signaling like p38 or ERK [38], [40], [41], [43]. However, SB-431542 can also block activin signaling but with a lower efficiency than TGF-β signaling as reported by the IC_50_ for ALK4 (activin type I receptor, IC_50_ = 140nM) and ALK5 (TGF-β type I receptor, IC_50_ = 94nM) [38], [41]. To investigate whether TGF-β signaling is required for limb regeneration, regenerating axolotls were treated with SB-431542. Results presented in Figure 4 show that limb regeneration was completely blocked in axolotls treated with SB-431542 at a final concentration of 25 µM (Fig. 4 A). This concentration represents the lower spectrum of concentrations reported in *in vivo* studies using this inhibitor [61]–[63]. At 25 µM, SB-431542 was not toxic for the animals as they grew and fed normally. In control animals, DMSO treatment did not prevent limb regeneration (Fig. 4 A). Treatment of axolotls with SB-431542 at lower concentrations provided incomplete blocking of regeneration (data not shown). When axolotls were treated with SB-431542, from the moment of amputation until control animals had regenerated (30 days), regeneration was completely blocked. In these animals, wound epithelium formation was observed but blastema formation was absent (Fig. 4 A). Animals were also treated with SB-431542 starting at early bud (EB) stage until the end of regeneration. When treated at EB, the blastema increased in size for a few days until it reached a final shape/size comparable to a late bud blastema, stopped growing and never regenerated (Fig. 4 A). The effects of SB-431542 treatments on regenerating tissue were also tested to determine whether the observed inhibition was reversible or permanent. Figure 4 B shows that limbs treated daily with SB-431542 for 7 or 14 days post-amputation did not reinitiate the regeneration process when the treatment was stopped. Animals treated with SB-431542 for the first 48 hours post-amputation regenerated their limbs with only a slight delay compared to controls (data not shown).

### Histological analysis of SB-431542 effect on limb regeneration

**Figure 5:**
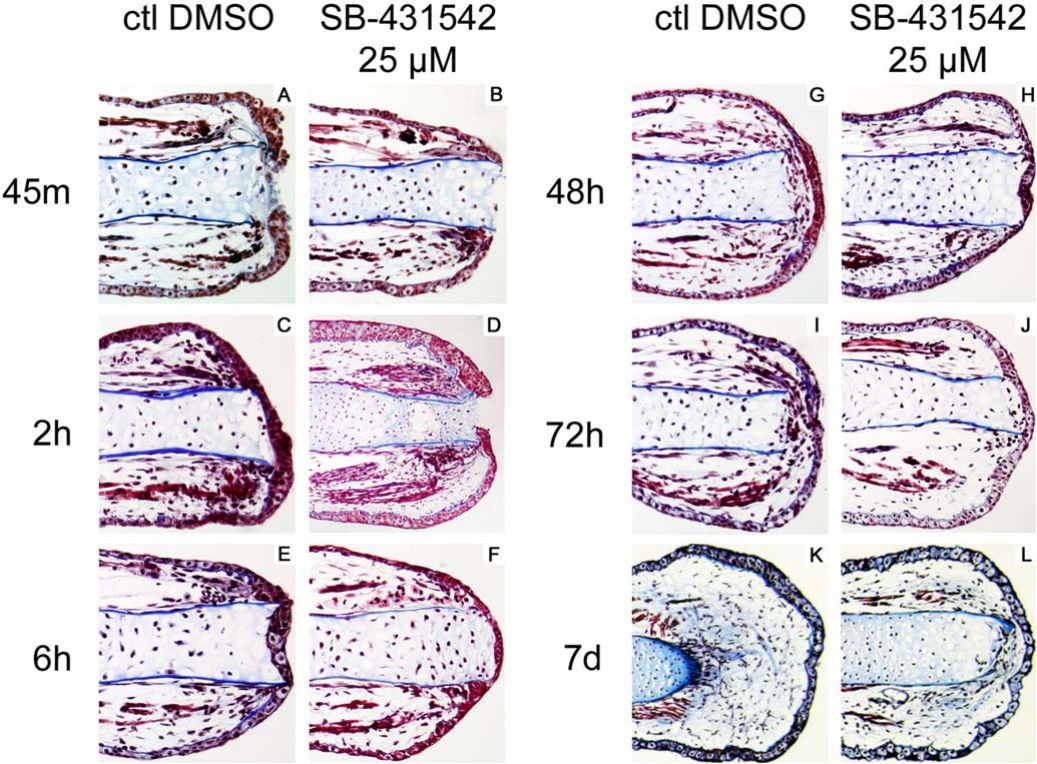
Histological analysis of regenerating limbs treated with SB-431542. Control limbs were treated with DMSO from time of amputation and fixed A) 45 minutes, C) 2 hours, E) 6 hours, G) 48 hours, I) 72 hours and K) 7 days after amputation. Samples treated with 25 µM SB-431542 from time of amputation were fixed at B) 45 minutes, D) 2 hours, F) 6 hours, H) 48 hours, J) 72 hours and L) 7 days after amputation. Masson's trichrome staining method was used to stain cell cytoplasm in red, collagen in blue and nuclei in black. Note the delayed closure of the wound epithelium in SB-431542 treated limbs at 45 minutes and 2 hours post-amputation. Also note that there is no blastema formation or accumulation of cells between the tip of the bone and the wound epithelium in SB-431542 treated limbs.

Figure 5 shows a time course of regenerating limbs treated with SB-431542 or DMSO (control). Although the wound at the tip of the stump is still open on both control and SB-431542 treated limbs, after forty-five minutes following amputation (Fig. 5 A and B), a slight delay in wound closure can be observed in SB-431542 treated limbs. Two hours after amputation, the WE had completely closed the wound in control limbs (Fig. 5 C) which is in accordance with results reported previously by Carlson *et al.* [14]. In SB-431542 treated limbs, the WE had not completely closed the wound 2 h post-amputation (Fig. 5 D). However, at 6 h post-amputation, the WE covered the tip of the limbs in both control and SB-431542 treated animals (Fig. 5 E and F). At 48 h post-amputation, streams of cells migrating between the end of the bone and the WE can be seen in controls but not in SB-431542 treated limbs (Fig. 5 G and H). At 72 h post-amputation, cells are still migrating toward the WE in control limbs (Fig. 5 I). Those cells will accumulate to form the regeneration blastema [1]. In limbs treated with SB-431542, no cellular accumulation at the end of the limb in any of the samples analyzed at 72 h could be observed (Fig. 5 J). At 7 days post-amputation, control limbs have reached early/medium bud stage with a prominent blastema (Fig. 5 K). Cells that have migrated and proliferated now occupy a large space between the tip of the bone and the WE. At this stage, the end of the bone in control limbs shows signs of being remodeled as previously reported by Tank *et al.* [64] (Fig. 5 K). In comparison, limbs treated with SB-431542 still show no sign of blastema formation and the tip of the bone did not show any sign of being remodeled as in control limbs at this time point (Fig. 5 L).

### Cell proliferation analysis in SB-431542 treated limbs

**Figure 6:**
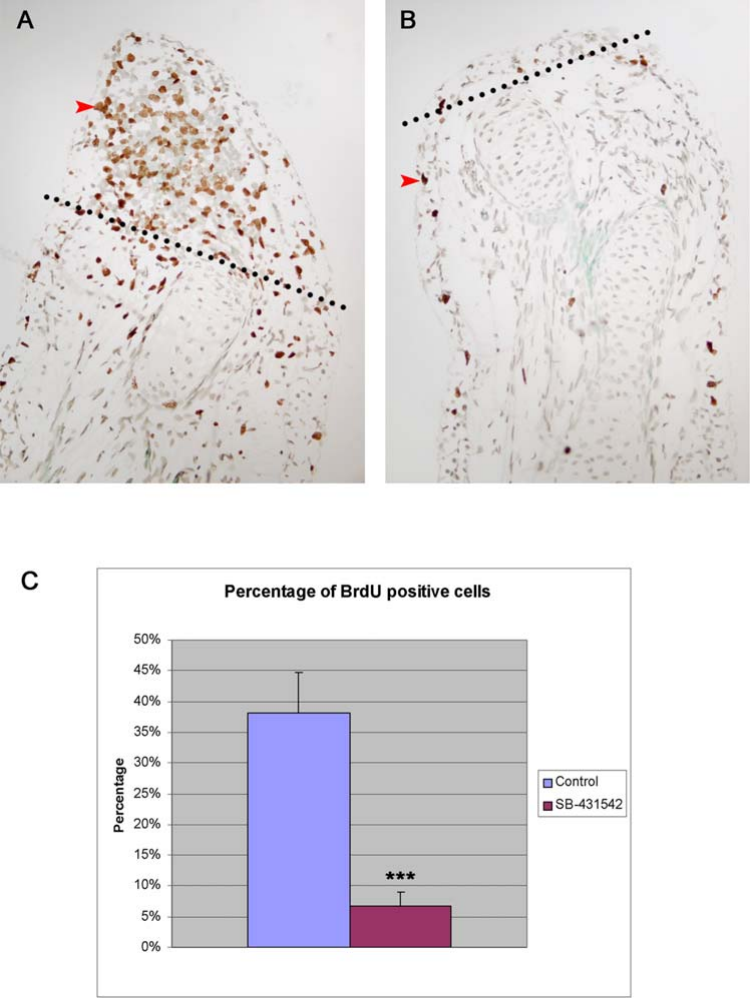
Inhibition of cellular proliferation in regenerating limbs treated with SB-431542. A) Control regenerating limb treated with DMSO and assessed for BrdU incorporation at 7 days post-amputation (medium bud stage). Red arrowhead marks a cell positive for BrdU. Note the accumulation of BrdU-positive cells in the regenerating blastema. Cells positive for BrdU are also found (at a lower frequency) in the epidermis of the non-regenerating part of the limb. B) SB-431542 treated limb assessed for BrdU incorporation 7 days post-amputation. No accumulation of BrdU-positive cells at the tip of the limb is observed. Only a few positive cells are found mostly in the epidermis of the limb (red arrowhead). Dotted lines in panels A and B represent the level of amputation. C) Graph comparing percentage of BrdU-positive cells in the regenerating blastema of control limbs (n = 3 animals) and in SB-431542 treated limbs (n = 3 animals). A statistically significant difference in the percentage of BrdU positive cells between control (38%±6.2%) and SB-431542 treated limbs (7%±2.1%) was observed (*** p<0.001).

Knowing that TGF-β signaling is important in regulating cell growth, the effect of SB-431542 treatment on cellular proliferation in axolotl limbs was verified. To measure this effect, a BrdU labeling experiment, consisting in injecting BrdU solution intra-peritoneally in control and SB-431542 treated axolotls, was performed 7 days post-amputation. Results presented in Figure 6 show BrdU labeled cells in the regenerating blastema of a control animal (Fig. 6 A) and in the limb of an SB-431542 treated animal (Fig. 6 B). In the control limb, many cells in the blastema are in a proliferative state as determined by the presence of BrdU labeled cells (Fig. 6 A). In comparison, a very small number of cells, excluding a few skin cells, are labeled for BrdU in SB-431542 treated limbs (Fig. 6 B). The graph presented in Figure 6 C shows a significant difference in the percentage of BrdU positive cells between the blastema region in controls and the tip of the limb in SB-431542 treated animals. These results indicate that SB-431542 blocks cellular proliferation in axolotls and suggest that TGF-β signaling controls cellular proliferation during limb regeneration.

### Measurement of TGF-β1 target genes expression in SB-431542 treated limbs

**Figure 7:**
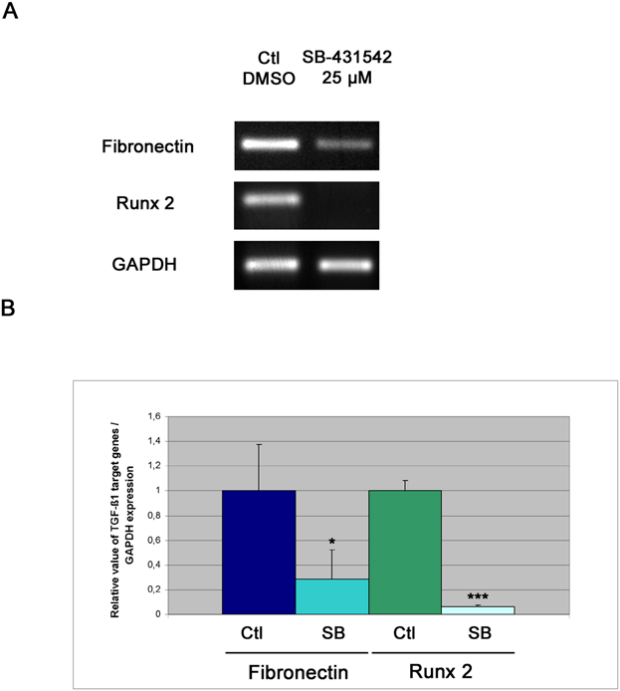
Inhibition of TGF-β1 target genes expression in regenerating limbs treated with SB-431542. A) RT-PCR showing expression of fibronectin and Runx 2 in axolotl regenerating forelimbs. RT-PCR reactions were performed on at least 4 separate RNA samples extracted from pools of 6 blastemas of animals treated with DMSO or SB-431542. Fibronectin and Runx 2 were strongly expressed in control limbs and significantly down-regulated in limbs treated with SB-431542. GAPDH was used as a control. B) Graph representing the relative value of fibronectin/GAPDH and Runx 2/GAPDH expression in control and SB-431542 treated limbs. Fibronectin and Runx 2 relative expression in control limbs were fixed to 1±0.37 and 1±0.09 respectively. The relative expression values in SB-431542 treated limbs were 0.29±0.23 with a p<0.05 (*) for fibronectin and 0.07±0.01 with a p<0.001 (***) for Runx 2.

After examining the effects of SB-431542 on cellular growth *in vivo*, the ability of this inhibitor to block the expression of two TGF-β1 target genes, fibronectin and Runx 2, during limb regeneration was tested. Figure 7 shows RT-PCR amplification of TGF-β1 target genes in control and SB-431542 treated limbs. Strong fibronectin and Runx 2 expressions were observed in control limbs 5 days after amputation (Fig. 7 A), which are consistent with the time-frame of studies looking at the expression of these genes in axolotl regenerating limbs [15], [65]. In contrast, both fibronectin and Runx 2 were significantly down-regulated in SB-431542 treated limbs (Fig. 7 A). GAPDH expression was not affected by SB-431542, as all other control genes tested (cyclophilin D, beta-actin, EF1-α, data not shown). Densitometric analysis of the PCR bands in Figure 7 A shows that fibronectin expression in control limbs was about 3 times higher and Runx 2 expression was about 15 times higher than in SB-431542 treated limbs (Fig. 7 B). These results demonstrate that SB-431542 treatment affects the expression of known TGF-β1 target genes, fibronectin and Runx 2, by down-regulating their expression in regenerating limbs.

## Discussion

TGF-β1 is a multi-functional cytokine implicated in many aspects of mammalian wound healing and scar tissue formation processes [5], [27], [66]–[69]. Interestingly, urodele amphibians such as the axolotl can regenerate many parts of their body following amputation and never seem to form scar-tissue [1]. Given that mammalian wound healing and axolotl limb regeneration share many similarities in their initial phases [2], [5], [10], we examined the requirement of TGF-β signaling and TGF-β1 expression during axolotl limb regeneration.

Isolation of the axolotl TGF-β1 cDNA and translation of the protein sequence through NCBI BLAST (http://www.ncbi.nlm.nih.gov/BLAST/) indicated that the sequence has high homology with mammalian sequences for TGF-β1 [i.e. very low (E) values (the probability due to chance, that there is another alignment with a greater similarity), the lowest (E) being 7e^−112^ and corresponding to *Equus caballus* (horse) TGF-β1]. These values confirmed that the isolated sequence corresponded to the axolotl TGF-β1. The sequence also has high homology with the TGF-β5 sequence of *Xenopus laevis,* an anuran amphibian [(E) value of 1e^−103^]. A TGF-β1 orthologue has never been isolated in *Xenopus laevis* for which over 650 000 EST are available (data from GenBank). However, phylogenetic analyses suggest that the *Xenopus* TGF-β5 is actually the orthologue of the mammalians TGF-β1 [23].

A rapid up-regulation of TGF-β1 expression was observed following limb amputation and was maintained at a high level throughout the preparation phase of limb regeneration. As mentioned above, TGF-β1 is a key regulator of wound epithelium formation, inflammation, blood clot formation, and ECM remodeling in mammalian wound healing processes [5], [26], [27], [32], [33]. Axolotl limb regeneration shares many of these processes with mammalian wound healing and most of them are essential for regeneration to take place [2], [8]. Fibroblasts participate in both processes: in mammals they invade granulation tissue and mediate wound contraction; in the axolotl they dedifferentiate and provide one of the main sources of blastema cells for the regenerating limb [2], [5], [35], [36]. At the early bud stage, when blastema cells are proliferating rapidly, a strong expression of TGF-β1 is present in the blastema. It is possible that, in the preparation phase of regeneration, TGF-β1 could be expressed to promote detachment and migration of mesenchymal cells towards the blastema. Martin *et al.* suggested that TGF-β1 acts in a similar way to promote mesenchymal cell proliferation during mammalian embryonic wound healing [70]. Mammalian embryos can heal their skin wounds perfectly following injury [71]. In E11.5 mouse embryos, TGF-β1 is rapidly expressed at the site of injury in response to wounding. TGF-β1 expression reaches a peak at 3–6 hours after wounding and is quickly down-regulated 18 hours post-wounding, just before the wound has fully closed/healed [70]. Moreover, just as in the axolotl, mammalian embryos present a diminished immune response following injury [5], [71], [72]. In mammalian adults, TGF-β1 rapidly promotes inflammation and recruits immune cells like macrophages, leukocytes and lymphocytes, when released at the wound site [45], [66], [73], [74]. Because of its pro-inflammatory effects, prolonging TGF-β1 expression at the wound site does not benefit wound healing [73]. However, it has been reported that axolotls are immuno-tolerant and exhibit a weak immune response following grafts or injuries [9], [75], [76]. In fact, very low levels of inflammatory cells are present at the wound site of amputated limbs [77]. It is believed that the reduced immune response following limb amputation could be linked to the regenerative capacity of salamanders [9]. The data presented in this paper demonstrate that TGF-β1 is expressed during the preparation phase of limb regeneration, when cells of mesenchymal origin dedifferentiate, migrate and divide rapidly to form the regeneration blastema.

In order to functionally analyze the role of TGF-β signaling during axolotl limb regeneration, we treated regenerating axolotls with the pharmacological inhibitor SB-431542. Numerous studies have reported the ability of SB-431542 to inhibit TGF-β signaling by blocking the ALK5 mediated phosphorylation of Smad2/Smad3 in various cell lines [38], [40]–[44], [78], [79]. Our data demonstrate that SB-431542 blocks axolotl limb regeneration and thus provides the first line of evidence that TGF-β signaling is essential for this process to take place. Histological analysis of SB-431542 treated limbs suggests that TGF-β signaling affects cellular migration during regeneration, since wound healing is delayed and blastema formation is absent in these limbs. TGF-β1 could be a key player in regulating this process as it is a potent chemo-attractant of fibroblasts during mammalian wound healing [30], [31]. TGF-β signaling also promotes cell growth in regenerating limbs as demonstrated by the very low incorporation of BrdU in SB-431542 treated limbs. The inhibitory effects of SB-431542 on limb regeneration could also in part be due to the down-regulation of fibronectin or Runx 2 expression. Fibronectin, a component of the extra-cellular matrix which is a target gene of TGF-β1, has been suggested to play an important role in epithelio-mesenchymal interactions during axolotl limb regeneration [15]. If those interactions do not take place or are reduced, limb regeneration could be inhibited [8], [16]. Runx 2, also named *Cbfa-1*, is a transcription factor that regulates mesenchymal condensation, chondrocyte hypertrophy and osteoblast differentiation [80], [81]. It is a target gene of TGF-β1 and was shown to be expressed in regenerating axolotl limbs [65], [82]. Inhibition of TGF-β signaling with SB-431542 in axolotl limbs resulted in a down-regulation of Runx 2 mRNA as previously shown in mammalian cells, [83]. Results also show that, beyond a certain point, the effect of SB-431542 treatment on regenerating limbs becomes irreversible. 48 hours of treatment with SB-431542 only slightly delayed limb regeneration (data not shown), but inhibition became irreversible when treatment was sustained for 7 days or more. The reasons why 7 days of treatment become irreversible are not clear at the moment. Histological analysis does not show any sign of scar tissue appearance like those reported by Odelberg's group while blocking MMPs [17]. It could be that the WE lost its permissive abilities due to the lack of mesenchymal cell accumulation to form a blastema. The present results obtained with SB-431542 imply that TGF-β signaling controls the formation and growth of the regeneration blastema. In mammals, wound healing often results in scar formation which is regulated by TGF-β signaling [27], [69], [84]. Many studies have reported reduction of scarring in mammals when wounds are treated with chemical inhibitors or specific antibodies aimed at TGF-β isoforms (particularly TGF-β1) during the healing process [66]–[68], [85]–[89]. The situation seems to be very different in the axolotl as blocking of TGF-β signaling prevented limb regeneration. Our data show that TGF-β signaling is essential for the regeneration of the axolotl limb, a complex tri-dimensional structure, and suggest a new role for TGF-β signaling in regenerative biology.

Recently, Jazwinska *et al.* used SB-431542 to examine zebrafish fin regeneration and reported that SB-431542 inhibited blastema formation in amputated fins, allowing only WE formation [90]. These results are consistent with our data in axolotls. In their paper, Jazwinska *et al.* demonstrate that SB-431542 blocked cellular proliferation in treated fins, as detected by a reduction of BrdU labeling which we also observe in axolotl limbs. Moreover, in both models, treatment of regenerating appendages at mid-stages of regeneration (4 days post-amputation in zebrafish and early bud stage in the axolotl) completely blocked the process, suggesting that TGF-β/activin signaling is essential to maintain the proliferation of blastema cells. In the present paper, whole-mount *in situ* hybridization and Northern blot results demonstrate that TGF-β1 is regulated during axolotl limb regeneration. Although Jazwinska *et al.* show that activin-βA (Act-βA) is the only TGF-β family member strongly regulated during zebrafish fin regeneration, their data also indicate that TGF-β1 and TGF-β3 are expressed at significant levels during the regeneration process [90]. Interestingly, their results indicate that TGF-β1 expression is between 10–40 times higher than that of Act-βA in uninjured fins and at 6h and 24h post-amputation. Considering the fact that SB-431542 has a higher affinity for the TGF-β type I receptor ALK-5 (IC_50_ = 94nM) than for the activin type I receptor ALK-4 (IC_50_ = 140 nM) [38], [41], the inhibition of zebrafish fin regeneration observed by Jazwinska *et al.* [90] could in part be due to the inhibition of TGF-β signaling.

In summary, we show that TGF-β1, a key player in mammalian wound healing, is rapidly up-regulated following amputation and strongly expressed during the preparation phase of axolotl limb regeneration. By blocking limb regeneration with SB-431542, a potent inhibitor of TGF-β type I receptor (ALK-5), we show that TGF-β signaling is essential for limb regeneration. The cellular mechanism by which TGF-β may regulate axolotl limb regeneration is an interesting area for future investigations and will help in understanding the differences and similarities between mammalian wound healing and axolotl tissue regeneration.

## Materials and Methods

### Animal maintenance and treatments

Axolotl (*Ambystoma mexicanum*) embryos and larvae were purchased from the Ambystoma Genetic Stock Center (Lexington, KY). Animals were maintained as previously published by Lévesque *et al*. [91]. SB-431542 (Tocris Bioscience, Ellisville, MO) was purchased from Cedarlane Laboratories (Burlington, ON) and diluted to a stock concentration of 10mM in DMSO. After limb amputation, animals (2–5 cm) were kept in 5 mL of 20% Holtfreter's solution containing SB-431542 at a final concentration of 25 µM or DMSO for controls. The Holtfreter's solution containing either SB-431542 or DMSO was changed daily. A minimum of 3 animals were treated for each condition. Animal care and experiments were done in accordance with the Université de Montréal animal care committee's guidelines.

### Cloning of axolotl TGF-β1 cDNA

Total RNA was extracted from axolotl larvae and cells using Trizol reagent (Invitrogen, Carlsbad, CA). Reverse transcription reactions were done at 50°C using Superscript II reverse transcriptase (Invitrogen). A 495 base pair cDNA fragment encoding the axolotl TGF-β1 was first isolated using RT-PCR from larvae total RNA with primers DFTGFB1 (degenerate forward TGF-β1) and DRTGFB1 (degenerate reverse TGF-β1) obtained from Sigma Genosys (Oakville, ON.). This fragment was then used to screen a pre-made axolotl cDNA library (cat. # 937670, Stratagene, La Jolla, CA). This screening resulted in the isolation of a larger fragment corresponding to the axolotl TGF-β1. To complete the sequence, 5′ RACE-PCR (Rapid Amplification of cDNA Ends) was done using the BD Smart-RACE kit (cat. # 634914, BD Biosciences, Mississauga, ON.). For this method, axolotl gene-specific primers were designed: ATGFR210 (Axolotl TGF-β1 reverse 210) and ATGFR367 (Axolotl TGF-β1 reverse 367). RNA from limb medium bud blastemas was used to synthesize the first strand for the 5′ RACE reaction. The RACE and PCRs were performed following the manufacturer's instruction. A PCR was performed to amplify the axolotl TGF-β1 complete cDNA using the following primer pairs: ATB1F1067 with ATB1R2419 which gave a fragment of 1352 base pairs, and ATB1F1098 with ATB1R2368 which gave a fragment of 1270 base pairs. These fragments were cloned into the PCR II-TOPO vector (Invitrogen). Following sequencing of each fragment at the Genome Québec sequencing Center a BLAST search was performed in GenBank (http://www.ncbi.nlm.nih.gov/BLAST/). The axolotl TGF-β1 protein structure domains were also obtained from GenBank (http://www.ncbi.nlm.nih.gov/Structure/cdd/cdd.shtml). The alignment of TGF-β1 protein sequences was compiled with the Lasergene program (DNAStar, Madison, WI) by using the Clustal W method. Human TGF-β1 sequence (accession number P01137), mouse TGF-β1 (accession number NP_035707) and *Xenopus laevis* TGF-β5 (accession number AAB64441) were aligned with the axolotl TGF-β1 sequence (accession number EU147783).

### Whole-mount *in situ* hybridization

Whole-mount *in situ* hybridization was performed as described by Gardiner *et al*. [7] with a few modifications. Digoxygenin labeled antisense RNA probe for TGF-β1 was synthesized using T3 RNA polymerase (Promega, Madison, WI) and DIG RNA labeling mix (Roche Diagnostics, Laval, QC). The PCR-Script Amp SK+ vector (Stratagene) containing the 495 bp TGF-β1 fragment was linearized with EcoR1 and used as template for probe synthesis. For tissue permeabilization, limbs were incubated with 30µg/ml proteinase K for 30–60 minutes on ice and then at 37°C for 45–60 minutes with the time adjusted for blastema size. Prehybridization and hybridization (72 hours) temperature was 62.5°C. For alkaline-phosphatase reaction, BM purple (Roche) was used as the enzyme substrate for the colorimetric reaction. No signal was detected at any stage when the sense probe was used for *in situ* hybridization (data not shown). A minimum of 3 samples, for each regeneration stage, was used for Whole-mount *in situ* hybridization.

### Northern blots

Limb regeneration stages were determined as described by Tank [64] and Iten [92]. Total RNA was extracted from blastemas at each stage using Trizol. T = 0 corresponds to RNA extracted from mature limb tissue. Messenger RNA was extracted using GenElute mRNA Miniprep kit (Sigma-Aldrich, St-Louis MO). 20 µg of total RNA or 5 µg of mRNA were loaded per lane on a 1% agarose-6.9% formaldehyde gel. RNA was transferred from the gel to a nylon membrane (Pall, USA) by capillary transfer. A cDNA probe coding for the axolotl TGF-β1 was amplified from the PCR-Script amp SK+ vector. The membranes were hybridized with this cDNA probe labeled with [α-^32^P]deoxy-CTP by the random hexamer method as in Roy *et al.* [93]. Northern blots were repeated 3 times with different RNA samples for each regeneration stage.

### Affinity labeling of axolotl cells

Axolotl cells (AL-1 cell line) grown in culture were maintained at a constant temperature of 26°C without CO_2_ in 60% Leibovitz's L-15 medium with 2mM L-glutamine, 100 units/mL penicillin-streptomycin, 1× insulin-transferrin-selenium (diluted from 100× stock solution, Invitrogen) and 5% fetal bovine serum (FBS; Gibco, Invitrogen). Axolotl cells in culture were labeled with [^125^I]-TGF-β1 human recombinant protein and immuno-precipitations of TGF-β receptors were performed, as described previously [50], [60]. Immuno-precipitations of TGF-β receptors were done using the following antibodies: anti-TGF-β RI (V-22) (Santa Cruz biotechnology, CA), anti-TGF-β RII (C-16) (Santa Cruz biotechnology), anti-betaglycan Get-1 as described in Piek *et al.* [52]. SDS-PAGE was performed under reducing conditions using β-mercaptoethanol. Affinity labeling of axolotl cells was repeated two times.

### UV irradiation of axolotl cells and Western blot analysis

Axolotl AL-1 cells were irradiated with ultra-violet (UV) light (500 J/m^2^) and were harvested 6, 12 and 24 hours post-irradiation. Total proteins were extracted by sonicating cells in sodium dodecyl sulfate (SDS) sample buffer and heated in boiling water 5 minutes before electrophoresis on 12% polyacrylamide-SDS gels according to the method of Laemmli [94]. Fifty micrograms of proteins were loaded per lane. The proteins were transferred electrophoretically onto PVDF membranes (Immobilon-P, Millipore, Bedford, MA). For Western blotting, we used a 1∶200 dilution of an anti-TGF-β RII (C-16) #sc-220 antibody (Santa Cruz biotechnology). Immuno-detection of primary antibody was visualized using the ECL Western blotting kit according to the manufacturer's directions (GE Healthcare, Buckinghamshire, UK). Experiments were repeated at least 3 times with different protein samples for each experiment.

### Treatments of axolotl cell line with TGF-β1 protein and SB-431542

Human recombinant TGF-β1 protein was used to treat AL-1 cells at a final concentration of 25 or 100 pM. Cells were serum starved and kept in 60.0% Leibovitz's L-15 medium for 48 h prior to adding TGF-β1. Total RNA was collected (using Trizol reagent) from AL-1 cells treated with TGF-β1 after 3 and 72 hours. HaCaT human keratinocyte cell line was grown in DMEM medium with 2mM L-glutamine, 100 units/mL penicillin-streptomycin, and 10% FBS and used as control. SB-431542 was added 30 minutes before TGF-β1 treatment. Each experiment was performed in triplicates.

### RT-PCR

RT-PCR reactions were done using RNA collected from axolotl cells in culture or regenerating limb blastemas. 30 cycles of PCR amplification were done for each gene. Primers used to amplify each genes are as follows: axolotl plasminogen activator inhibitor 1 (PAI-1) APAI1F183 and APAI1R706, axolotl fibronectin AFNF31 and AFNR411, axolotl Runx 2 ARUNX2F66 and ARUNXR295, human fibronectin HFNF87 and HFNR335, axolotl glyceraldehyde-3-phosphate dehydrogenase (GAPDH) AGDHF90 and AGDHR391 and human glyceraldehyde-3-phosphate dehydrogenase HGAPDH 5′ and HGAPDH 3′ (See Table 1 for primer sequences). To measure the effect of SB-431542 on TGF-β1 target genes expression, animals were treated for 5 days following amputation with DMSO (controls) or SB-431542. RNA was extracted from blastemas or the tip of the limbs of comparable size for SB-431542 treated animals and RT-PCR was performed. Each sample comes from a reverse transcription reaction made with 6 blastemas from control or SB-431542 treated limbs. Student's T test was performed to confirm statistical significance of fibronectin and Runx 2 expression in control versus SB-431542 treated limbs (4 samples per condition).

**Table 1:** Primers used in RT-PCR reactions

Primer	Sequence
DFTGFB1	TGG(G,C)TGTC(C,T)TT(C,T)GA(C,T)GT(C,T)AC
DRTGFB1	C(C,T)GGGTTGTG(A,C)TG(G,C)TT(A,G)TAC
ATGFR210	CGGTGGTCACAGTTACGAGGAA
ATGFR367	CACACTGATACAGCTCCACTCG
ATB1F1067	TCGTTGGCCCTATATTGTCC
ATB1R2419	CTCCCATTGCCTTTACTCGT
ATB1F1098	CTCGGTTGAGGGAACTCTTG
ATB1R2368	CAGTTCCATTCGCTTTGACA
APAI1F183	TCCCTCTGACCACCTTGACT
APAI1R706	CTCGAGGAAGGGTTGAGAGA
AFNF31	AGGAGATCTGCACCACCAAC
AFNR411	TCTCCCGGCCATAACAGTAG
ARUNX2F66	GCCTTCAAGGTGGTAGGTCTC
ARUNXR295	CTGTGGTAGGTGGCTACTTGG
HFNF87	CAGTGGGATAAGCAGCATGA
HFNR335	CTCTGAATCCTGGCATTGGT
AGDHF90	GACAAGGCATCTGCTCACCT
AGDHR391	ATGTTCTGGTTGGCACCTCT
HGAPDH 5′	ACCACAGTCCATGCCATCAC
HGAPDH 3′	TCCACCACCCTGTTGCTGTA

### Histology

Following treatments with SB-431542 or DMSO, axolotls were fixed overnight in Bouin's fixative solution and then rinsed thoroughly with 70% alcohol. Limbs were embedded in paraffin and cut to 10 µm sections. Slides were deparaffinized through 3 baths of toluene for 5 minutes each. Slides were then rehydrated in a graded series of 100%, 90%, 70% and 50% alcohol and then distilled water for 5 minutes each. For histology, Masson's trichrome staining method was used to stain cell cytoplasm in red, nuclei in black and collagen in blue [95]. A minimum of 3 samples were stained for each time-point.

### BrdU incorporation assay

Regenerating control and SB-431542 treated axolotls were injected intra-peritoneally, 7 days after amputation, with 10µL of BrdU stock solution using a ratio of 1–2mL/100 g of body weight according to the manufacturer's instructions (GE Healthcare, # RPN201). Animals were fixed 12 hours after injection in 4% paraformaldehyde in 0.7× PBS for 24 hours at 4°C. Samples were paraffin embedded, deparaffinized and rehydrated as for histology. For immuno-histochemistry, slides were washed 4×15 minutes in PBST (1× PBS with 0,1% Tween 20). They were incubated with 0.8% Pepsin in 0,2N HCl for 10 minutes at 37°C to promote denaturation of DNA. Slides are then washed 3×10 minutes in PBST, and incubated in a blocking solution (2% bovine serum albumin, 1% DMSO, 10% sheep serum and 0,1% Triton X-100) at room temperature for one hour. Slides were then incubated overnight at 4°C with an anti-BrdU mouse monoclonal antibody (BrdU Ab-3, Labvision/Neomarkers, Fremont, CA.) diluted 1:50 in blocking solution. PBST washes (4×15 minutes) were done before incubating with an anti-mouse secondary antibody coupled to horseradish peroxidase, dilution 1∶250, for 2 hours at room temperature (GE Healthcare). Slides were washed again 4×15 minutes in PBST before being incubated in DAB (Zymed, Invitrogen) for signal detection. Slides were counterstained with methyl green for 6 minutes (Dako, Mississauga, ON) before being serially dehydrated 2×2 minutes each in 90% EtOH, 100% EtOH and 100% Xylene. Slides were mounted with Permount (Fisher scientific, Ottawa, ON). Immunohistochemical detection for BrdU incorporation was done on three different samples. Student's T test was performed to confirm statistical significance of BrdU incorporation in control versus SB-431542 treated limbs (3 samples per condition).
